# Successful fresh formulation CD19 CAR-T cell therapy for GAD65 antibody-mediated cerebellar ataxia. A Case Report

**DOI:** 10.3389/fimmu.2026.1755797

**Published:** 2026-02-17

**Authors:** Mantas Vaisvilas, Skirmante Cernauskiene, David Petrosian, Natasa Giedraitiene, Mindaugas Stoskus, Laimonas Griskevicius

**Affiliations:** 1 Institute of Clinical Medicine, Faculty of Medicine, Vilnius University, Vilnius, Lithuania; 2 Hematology, Oncology and Transfusion Medicine Center, National Cancer Center, Vilnius University Hospital Santaros Klinikos, Vilnius, Lithuania

**Keywords:** autoimmune cerebellar ataxia, autoimmune encephalitis, CAR-T cell therapy, GAD65 antibody, gene therapy, immunotherapy

## Abstract

**Background:**

Chimeric antigen receptor T (CAR-T) cell therapy is an effective treatment for treatment-refractory hematological disorders with an acceptable safety profile. In contrast, preliminary reports suggest good efficacy for treatment-refractory autoimmune disorders, including autoimmune nervous system disease, but their safety profile is largely unknown.

**Objective:**

To describe the first case of glutamic acid decarboxylase-65 (GAD65) antibody-mediated cerebellar ataxia (CA) successfully treated with CD19 CAR-T cells.

**Results:**

A 33-year-old male was diagnosed with GAD65 antibody mediated CA in 2023. Despite treatment with Rituximab and Cyclophosphamide, the patient’s condition worsened with new-onset recurrent falls and increasing vertigo. Ambulation was maintained. CD19 CAR-T cells at a dose of 1 × 10^6^ cells per kilogram of body weight were infused after administration of standard lymphodepleting chemotherapy, resulting in a good serological response with reduction of GAD65 serum titers by 95% at day +90, significant clinical improvement in ataxia at day +30 and no evidence of disease progression at day +270 clinically, radiologically and laboratory-wise. The toxicity was limited to cytokine release syndrome grade 1.

**Discussion:**

The favorable clinical response observed in our patient, along with other reports demonstrating preliminary efficacy and limited toxicity, supports further study of CD19 CAR-T cell therapy in GAD65 neurological disorders.

## 1 Introduction

Glutamic acid decarboxylase 65 (GAD65) mediated cerebellar ataxia (CA) is a distinct autoimmune disease, characterized by high titers of GAD65 antibodies in the presence of gait-predominant and chronically progressive cerebellar syndrome (1). Several *in vitro* and *in vivo* studies support the pathogenic role of GAD65, demonstrating that the passive transfer of GAD65 antibodies produces pathogenic effects in rats, while the absorption of these antibodies leads to a full reversal of neuronal functionality (2, 3). Likewise, clinical studies suggest that high titers of GAD65 lead to poor outcomes in CA while reduction of antibody titers may be associated with clinical improvement (4). This suggests that antibody depletion may be effective for the treatment of GAD65 CA. There is no standard treatment for GAD65 CA, and despite the use of B-cell directed therapies, long-term outcomes in GAD65 CA are unfavorable in two-thirds of patients (5). An increasing number of reports suggest CAR-T cells may be a safe and effective therapy for treatment-refractory autoimmune diseases, including various immune-mediated neurological disorders. However, their safety and efficacy have been studied in a very limited number of immune-mediated neurological disorders. Two case reports of standard treatment-refractory GAD65-associated stiff person syndrome treated with CD19 CAR-T cells showed good response to treatment (6, 7). There are no reports of CD19 CAR-T cell therapy use in GAD65 CA. Herein, we describe a patient with GAD65 CA treated with CD19 CAR T cells.

## 2 Materials and methods

Case description with prospective follow-up over a 9-month period using pre-established follow-up protocols (**Supplementary Document S1**, **Supplementary Table S2**). Figures were generated using Python (version 3.11.4) and Adobe Photoshop (version 26.11, 2025).

## 3 Case report

### 3.1 Case description

**Figure 1:**
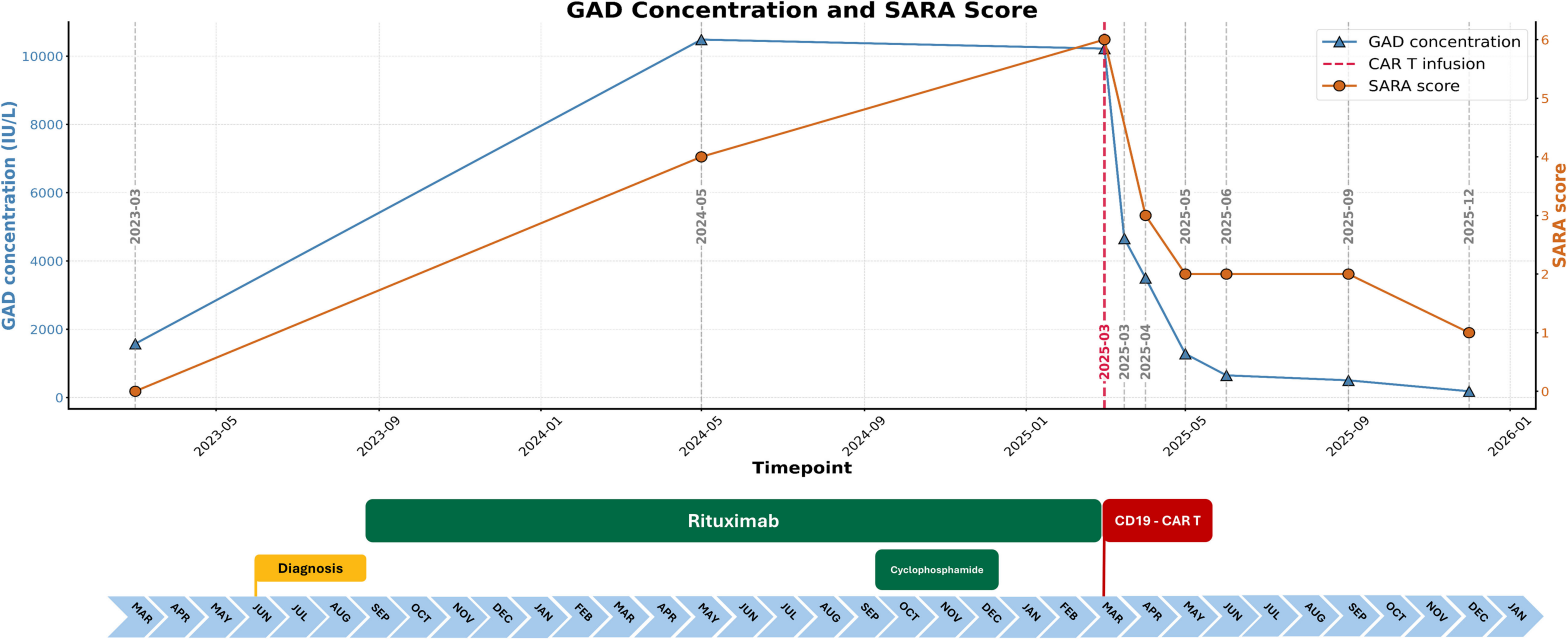
Disease evolution. The figure shows the progression of disease from diagnosis to the final follow-up. The temporal relationship between the disease course, treatment modalities, clinical severity, and the levels of serum GAD65 antibodies is shown.

A 33-year-old male was diagnosed with GAD65 CA in 2023 based on progressive central vertigo and high serum and cerebrospinal fluid (CSF) titers of GAD65 antibodies (detailed clinical information is available in **Supplementary Table S1**; **Figure 1**). Despite treatment with a combination of Rituximab and Cyclophosphamide, the patient’s condition worsened with new-onset, recurrent falls and increasing vertigo. Ambulation was maintained.

### 3.2 CD19 CAR-T cell therapy

**Figure 2:**
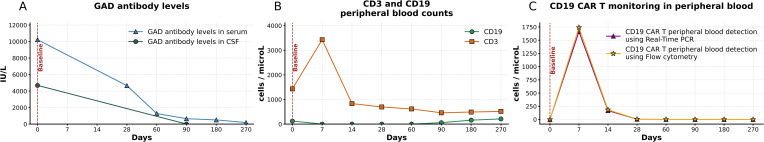
B, T cell counts, GAD65 dynamics and CD19 CAR-T cell expansion parameters. “Baseline” refers to laboratory parameters measured prior to lymphodepletion. X-axis shows time in days following CD19 CAR-T infusion (Baseline). **(A)** Line plot showing baseline GAD65 serum and CSF concentrations and their levels following CD19 CAR-T infusion. **(B)** CD3 and CD19 counts prior to and after CD19 CAR-T infusion in peripheral blood. **(C)** CD19 CAR-T cell counts in peripheral blood using different detection techniques.

In hopes of maintaining ambulation, cerebellar reserve and preventing the development of permanent CA, after obtaining patient’s informed consent, in-house CD19 CAR-T cells (detailed information regarding patient screening, CD19 CAR-T manufacturing, lymphodepletion, assessment of adverse events and follow-up monitoring are presented in **Supplementary Document S1**) at a dose of 1 × 10^6^ cells per kilogram of body weight were infused after administration of standard lymphodepleting chemotherapy (**Supplementary Document S1**). The treatment was approved by Ethics Committee of Vilnius University Hospital Santaros Klinikos. Rapid CAR-T cell expansion on day +7 resulted in transient CD19/20 cell peripheral blood aplasia from day +7 to day +90. (**Figure 2**). CD3/4+, CD3/CD8+ cell populations remained unaffected throughout the follow-up. Both peripheral and CSF circulating CD19 CAR-T cells were no longer detectable at day +90.

### 3.3 Follow-up

The patient was followed for 9 months. Standardized clinical, laboratory and radiological parameters (**Supplementary Table S2**) were measured in 1–3 months intervals to determine clinical and laboratory response, monitor potential adverse events, and assess the persistence of circulating CD19 CAR-T cells and B-cell aplasia.

### 3.4 Safety

Infusion-related side effects were limited to grade I cytokine release syndrome on day +1, with recurrent fever of >38.0 °C, which completely resolved after a single infusion of 640 mg of Tocilizumab. IgG levels remained unaffected at all time points throughout the follow-up. No serious infections requiring systematic antimicrobial therapies, Cytomegalovirus or Epstein-Barr virus reactivation were documented throughout the follow-up period.

### 3.5 Outcome measures

We observed a good serological response with a reduction of GAD65 serum titers by 95% at day +90, which continued to decrease despite the loss of B-cell aplasia. The GAD65 CSF titers became negative at day +90. Ataxia measurements improved at day +30 with subjective improvement of balance and vertigo and objective improvement in the Scale for the Assessment and Rating of Ataxia (SARA) scale (**Figure 1**, **Supplementary Table S2**). SARA scores, 9-Hole Peg Test (9-HPT) and 5.5-meter walking test measures did not show evidence of disease progression at +270. Cerebellar hemisphere and vermian structural magnetic resonance imaging (MRI) performed at baseline, 3, 6 and 9 months after CAR T infusion showed no evidence of atrophy (**Supplementary Table S2**).

## 4 Discussion

In this report, we present the first case of treatment-refractory GAD65 antibody-mediated CA successfully treated with CD19 CAR-T cells. Over a follow-up period of 9 months, we observed a favorable toxicity profile and no treatment-related serious adverse events. Additionally, we documented a rapid and sustained serological response, with a 95% reduction in serum GAD65 antibody levels from baseline and seroconversion to negative in the CSF, along with no evidence of disease progression as indicated by various clinical and radiological parameters.

Although the antigenic target for GAD65 antibodies is intracellular, supporting T cell-mediated pathogenesis, previous studies on GAD65 neurological syndromes suggest that B cells are pivotal early in the GAD65-mediated disease course to maintain T cell autoreactivity (8, 9). Likewise, *in vitro* evidence suggests that T cell autoreactivity is upregulated by B cells in germinal centers within the CNS (10). Eliminating B cells within tissue-resident lymphoid follicles may therefore halt T-cell-mediated tissue injury. This hypothesis is further corroborated in reports of successful treatment of T cell-mediated diseases with CD19 CAR-T cells (11, 12). In the present context, the effectiveness of CAR T cells in treating CNS neurological disorders is attributed to their ability to penetrate the CNS (13) and modify the interaction between B and T cells through B cell depletion. This is further supported by the limited effectiveness of standard anti-CD20 monoclonal antibodies in treating GAD65-mediated neurological disorders, as these antibodies have difficulty penetrating the CNS.

The timing of treatment is an important consideration in GAD65 antibody-mediated neurological disorders. The limited therapeutic response, even to CAR-T cells in some earlier reports (6, 7) suggests that symptoms may be reversible due to inhibition of neuronal function in the early course of the disease, but a time-dependent irreversible loss of GABAergic or Purkinje neurons follows (8, 9). Likewise, previous studies have shown that treatment for CNS T cell–mediated diseases, including CA, is effective only when administered early and in patients with retained ambulation (14, 15). The early therapeutic intervention is also supported by a recent expert opinion statement suggesting that early election of CD19 CAR-T may have beneficial effects across the entire spectrum of immune-mediated neurological disorders (16), including Diacylglycerol lipase alpha (DAGLA) antibody-associated ataxia-encephalitis, a novel autoimmune disorder that is most likely T cell-mediated (17). After thorough discussion with the patient, and encouraged by recent CAR-T studies in autoimmune diseases demonstrating limited adverse events (18) we elected to administer CD19-directed CAR-T cells early in the course of CA.

Although we did not observe acute or delayed immune effector cell-associated neurotoxicity syndrome (ICANS) in our case, available data from phase I trials suggest ICANS may still develop in a small fraction of patients treated with CAR-T cell therapy (19–21). In contrast to the hematological population, algorithms to identify patients at high-risk for ICANS in the autoimmune population are lacking, complicating patient selection and potentially compromising safety. Future trials will answer these important questions. Likewise, histopathological studies of ICANS cases are essential for understanding potential mechanisms of CAR-T cell-related toxicities in oncologic as well as autoimmune populations (22).

A potential limitation of the study is that we did not perform CSF measurements early after CD19 CAR-T infusion. This limited our ability to evaluate the capacity of intrathecal CD19 CAR-T cell expansion.

Likewise, in contrast to previous reports of CD19 CAR-T cells showing a dramatic improvement of severe neurological burden in stiff person syndrome and autoimmune encephalitis (7, 12, 17), our patient had minor neurological disability resulting in minor improvement. However, this minor improvement is likely clinically significant, as it surpasses the minimal clinically important difference (MCID) of the SARA score by a factor of two, as reported in previous ataxia studies (23). Lastly, the patient reported a subjective improvement in vertigo and balance after CD19 CAR-T cell therapy, while previous therapies with two second-line medications have failed to control the condition.

In summary, the favorable serological and clinical response in our patient, along with previous reports of efficacy, support further study of CD19 CAR-T cells for GAD65-related neurological syndromes in carefully selected patients. Both short and long-term safety, patient outcomes and toxicity profiles must be studied in large-scale trials.

## Data Availability

The original contributions presented in the study are included in the article/**Supplementary Material**. Further inquiries can be directed to the corresponding author.
