# Supplementary material for: Successful fresh formulation CD19 CAR-T cell therapy for GAD65 antibody-mediated cerebellar ataxia. A Case Report
Source: Front Immunol. 2026 Feb 17;17:1755797. doi: 10.3389/fimmu.2026.1755797 (PMC12953506; doi:10.3389/fimmu.2026.1755797)
Supplement: Supplementary file 1 [file Table1.docx]

**eTable1.** Clinical information of the patient.

| Age at onset | 31 |
| --- | --- |
| Clinical syndrome | Central positional vertigo with transient recurrent spells of cerebellar ataxia |
| Time from symptom onset, to diagnosis, months | 11 |
| Total disease duration to present, months | 38 |
| Follow-up from diagnosis to present, months | 27 |
| Antibody detection methods | Rat brain immunohistochemistry confirmed by GAD65 specific CBA;  Both serum and CSF positive |
| Initial MRI | Normal, no cerebellar atrophy |
| CSF analysis | Cells: 5x10^6/L  Protein: 0.45 g/L  IgG index: 0.52  Oligoclonal bands: absent; |
| Treatment | Rituximab  Total dose: 4000 mg i/v;  Treatment duration: 15 months;  Cyclophosphamide  Total dose: 3000 mg i/v  Treatment duration: 3months; |

CBA-Cell-based assay using HEK293 cells overexpressing GAD65 protein; CSF- cerebral spinal fluid; IgG- immunoglobulin G subclass; MRI-Magnetic resonance imaging;
