# Supplementary material for: Successful fresh formulation CD19 CAR-T cell therapy for GAD65 antibody-mediated cerebellar ataxia. A Case Report
Source: Front Immunol. 2026 Feb 17;17:1755797. doi: 10.3389/fimmu.2026.1755797 (PMC12953506; doi:10.3389/fimmu.2026.1755797)
Supplement: Supplementary file 2 [file Table2.docx]

**Table S2.** Baseline and follow-up standartised clinical, laboratory and radiological parameters.

| Parameter | Baseline | Day +7 | Day +14 | Day +28 | Day +60 | Day +90 | Day +180 | Day +270 |
| --- | --- | --- | --- | --- | --- | --- | --- | --- |
| Serum parameters | | | | | | | |  |
| GAD serum IU/L ELISA | 10219 | NA | NA | 4657 | 1281 | 648,85 | 503,99 | 183.09 |
| CD19 cells/mm^3^ | 123 | 0 | 0 | 0 | 0 | 57 | 160 | 209 |
| CD3 cells/mm^3^ | 1431 | 3428 | 833 | 694 | 615 | 458 | 489 | 515 |
| CD3/CD4 cells/mm^3^ | 1043 | 1826 | 427 | 444 | 376 | 286 | 337 | 362 |
| CD3/CD8 cells/mm^3^ | 318 | 1416 | 384 | 234 | 220 | 158 | 135 | 132 |
| IgG g/L | 12.83 | 11.96 | 11.83 | 10.77 | 10.41 | 10.25 | 10.48 | 10.47 |
| IgM g/L | 0.27 | 0.26 | 0 | 0 | 0 | 0 | 0.49 | 0.3 |
| IgA g/L | 0 | 0 | 0 | 0 | 0 | 0 | 0 | 0 |
| CD19 CAR T (cells/mm3) detection in peripheral blood using Real-Time PCR | 0 | 1663.6 | 168.1 | 4.7 | 0.80 | 0.20 | <0.1 | 0 |
| CD19 CAR T (cells/mm3 ) detection in peripheral blood using Flow Cytometry | 0 | 1741.42 | 183.43 | 6.52 | 0.80 | 0 | 0.83 | 0.1 |
| Fraction of CD19 CAR T in CD3 lymphocytes, peripheral blood, % | 0 | 53 | 26 | 1.2 | 0.1 | 0.02 | 0 | 0 |
| CSF parameters | | | | | | | |  |
| GAD CSF IU/L ELISA | 4687 | NA | NA | NA | NA | 2.26  (negative) | NA | NA |
| Fraction of CD19 CAR T cells in CD3 lymphocytes, CSF, % | 0 | NA | NA | NA | NA | 0 | NA | NA |
| IgG index | 0.52 | NA | NA | NA | NA | NA | NA | NA |
| OCB | Absent | NA | NA | NA | NA | Absent | NA | NA |
| Cells | 5 | NA | NA | NA | NA | 3 | NA | NA |
| Cranial MRI imaging | | | | | | | |  |
| Atrophy | Absent | NA | NA | NA | NA | Absent | Absent | Absent |
| Signal abnormalities | Absent | NA | NA | NA | NA | Absent | Absent | Absent |
| Clinical scores | | | | | | | |  |
| 5-meter walking test, s* | 4.2 | NA | NA | 3.77 | 3.76 | 3.89 | 3.91 | 3.97 |
| 9HPT Right, s* | 19.53 | NA | NA | 16.02 | 16.46 | 16.39 | 19.45 | 17.14 |
| 9HPT Left, s* | 17.38 | NA | NA | 18.11 | 17.39 | 17.01 | 18.57 | 18.29 |
| SARA | 6** | NA | NA | 3 | 2 | 2 | 2 | 1 |

CAR T- chimeric antigen receptor T cell; CD-cluster of differentiation; CSF- cerebral spinal fluid; Cr- creatinine; ELISA- enzyme linked immunosorbent assay; GAD- glutamic acid decarboxylase; Ig- immunoglobulin; MRI- magnetic resonance imaging; NAA- N-acetylaspartate; OCB- oligoclonal bands; SARA- Scale for the Assessment and Rating of Aaxia; 9HPT- 9-hole peg test.
*Expressed in means.
****** Gait 1/8; Stance 1/6; Speech 1/6; Finger chase R/L 1/4; Nose finger test R/L 1/4; Heel-shin slide R/L 1/4.

**Description of methods:**

**Cranial MRI imaging**

All measurements have been evaluated by the same radiologist with the same hardware.

Hardware: Phillips Achieva 3T; Software: Release 5.

Structural imaging: T1W 3D, FLAIR, T2 TSE, VEN BOLD, DWI sequencing.

**Functional testing**

The same rater performed all tests at baseline and throughout the follow-up.

**5-meter walking test**

Performed in accordance to previously published guidelines (1); Expressed in mean calculated from 3 attempts; Reference value: <6s. considered normal.

**Scale for the assessment and rating of ataxia (SARA)**

Performed in accordance to previously published guidelines (2).

**9-hole peg test**

Performed in accordance to previously published guidelines (3). Expressed in mean calculated from 2 attempts in dominant (R) and non-dominant (L) hands. Reference values for age and sex (4): dominant <20 seconds; Non-dominant <27 s.

**Literature**

1. Bohannon RW. Comfortable and maximum walking speed of adults aged 20-79 years: reference values and determinants. Age Ageing. 1997;26(1):15-9.

2. Schmitz-Hubsch T, du Montcel ST, Baliko L, Berciano J, Boesch S, Depondt C, et al. Scale for the assessment and rating of ataxia: development of a new clinical scale. Neurology. 2006;66(11):1717-20.

3. Fischer JS, Rudick RA, Cutter GR, Reingold SC. The Multiple Sclerosis Functional Composite Measure (MSFC): an integrated approach to MS clinical outcome assessment. National MS Society Clinical Outcomes Assessment Task Force. Mult Scler. 1999;5(4):244-50.

4. Wang YC, Bohannon RW, Kapellusch J, Garg A, Gershon RC. Dexterity as measured with the 9-Hole Peg Test (9-HPT) across the age span. J Hand Ther. 2015;28(1):53-9; quiz 60.
