# Supplementary material for: Successful fresh formulation CD19 CAR-T cell therapy for GAD65 antibody-mediated cerebellar ataxia. A Case Report
Source: Front Immunol. 2026 Feb 17;17:1755797. doi: 10.3389/fimmu.2026.1755797 (PMC12953506; doi:10.3389/fimmu.2026.1755797)
Supplement: Supplementary file 3 [file Table3.docx]

**Screening Evaluation**

A comprehensive screening evaluation was conducted nine days prior to CAR T-cell administration to determine the patient’s eligibility for treatment. Demographic data and detailed medical history were collected, including information on the initial diagnosis, disease-associated symptoms and manifestations, all prior therapies for the underlying disease with corresponding treatment responses, as well as relevant past and concomitant medical conditions.

A complete physical examination was performed, and vital signs were recorded. Screening procedures included pulmonary function testing, chest radiography, echocardiography, and a 12-lead electrocardiogram (ECG). Serological testing was performed for HIV-1/2 antibodies, hepatitis B surface antigen (HBsAg), hepatitis B core antibody (HBcAb), hepatitis C virus (HCV) antibodies, and Treponema pallidum hemagglutination assay (TPHA).

**Safety laboratory assessments included the following:**

- Hematology: Full blood count (FBC) with differential and lymphocyte subset analysis (CD3, CD4⁺, and CD8⁺ T cells; B cells; and natural killer [NK] cells, including CD3 counts for leukapheresis).
- Clinical Chemistry: Serum levels of creatinine, urea, uric acid, calcium, magnesium, sodium, potassium, chloride, total protein, albumin, liver function parameters (total bilirubin, alkaline phosphatase, aspartate aminotransferase [AST], alanine aminotransferase [ALT], and gamma-glutamyl transferase [GGT]), B-type natriuretic peptide (BNP), lactate dehydrogenase (LDH), C-reactive protein (CRP), erythrocyte sedimentation rate (ESR), and ferritin.
- Coagulation Profile: Fibrinogen, partial thromboplastin time (PTT), and international normalized ratio (INR).
- Immunology: Serum immunoglobulin G (IgG), immunoglobulin A (IgA), and immunoglobulin M (IgM)concentrations.
- Urinalysis.

**CAR T cell manufacturing**

Leukapheresis was performed on Day −7 using the **Spectra Optia Apheresis System** (Terumo BCT Inc., Lakewood, CO, USA). The leukapheresis product was processed within 24 hours without cryopreservation using the **CliniMACS Prodigy** automated cell processing platform (Miltenyi Biotec, Bergisch Gladbach, Germany). CAR T cells were manufactured using a second-generation anti-CD19 **chimeric antigen receptor (CAR)** construct, consisting of a single-chain variable fragment (scFv) specific for CD19, linked to the **4-1BB** costimulatory domain and a **CD3ζ** activation domain. CAR T manufacturing **process** was completed over **six days**. **In-process control (IPC)** and **quality control (QC)** assessments were performed using the **MACSQuant Analyzer 10** (Miltenyi Biotec) on Day 0 (pre-transduction) and Day 6 (post-expansion). Safety testing —including **sterility**, **endotoxin**, and **mycoplasma DNA** assays—was conducted on both intermediate and final products. The normalized **mean vector copy number (mVCN)** was determined by **digital PCR (dPCR)**. The final product met predefined **release criteria** for sterility, mycoplasma DNA, and endotoxin levels and was released for clinical use in a **fresh (non-cryopreserved) formulation**.

**Lymphodepletion and CAR T cell infusion**

The patient received **lymphodepleting (LD) chemotherapy** consisting of **fludarabine 30 mg/m²** administered intravenously (i.v.) on **Days −5, −4, and −3**, and **cyclophosphamide 300 mg/m² i.v.** on the same days, prior to CD19 CAR T-cell infusion. On **Day 0**, the patient received the **autologous fresh CD19 CAR T cells** at a target dose of **1 × 10⁶ CAR-positive T cells per kilogram of body weight**. The product was administered as a **short intravenous infusion** following **premedication with antihistamines and acetaminophen** to minimize the risk of infusion-related reactions. Post-infusion, the patient received **oral seizure prophylaxis with levetiracetam for one month, antiviral prophylaxis with acyclovir** for two months and **antimicrobial prophylaxis with cotrimoxazole** for six months following CAR T-cell therapy.

**Adverse Event Assessments**

Physical examinations, including vital signs, were performed daily through Day 14 following CAR T-cell infusion during the inpatient period. Subsequent assessments were conducted at Weeks 4, 8, and 12, and then every three months thereafter. Concomitant medications were documented at each visit, and safety laboratory tests as described in the screening procedure were repeated.

Patients were monitored daily for the first 14 days for signs of cytokine release syndrome (CRS) and immune effector cell-associated neurotoxicity syndrome (ICANS), using the CARTOX Working Group consensus grading scales. To evaluate potential hematological toxicity, complete blood counts were measured daily for the first 14 days, then weekly until Month 1, monthly until Month 3, and subsequently every 3–6 months. Immune effector cell-associated hematotoxicity was assessed according to the EHA/EBMT consensus grading criteria. All other adverse events (AEs) were graded according to NCI Common Terminology Criteria for Adverse Events (CTCAE) version 5.0. All infections occurring after CAR T-cell infusion were documented and reported.

**Monitoring of CAR T Cells and Leukocyte Subsets**

**Flow cytometry** was used to assess **leukocyte populations**, while **CAR T-cell expansion** was quantified by both **flow cytometry** and **quantitative Real-time PCR**. Assessments were performed at the time of CD19 CAR T-cell infusion (Day 0), **weekly until Month 1**, **monthly until Month 3**, and thereafter **every 3–6 months**.
